# Gynaecology

**Published:** 1877-11

**Authors:** 


### I. Gynaecology.

Influence of Alcohol upon the Quality of the Nurse's Milk.—Verney. (*Jour. de Méd. et de Chir. prat.* Sept., 1877, p. 417.)—The author, in discussing the influence of alimentation upon the quantity and quality of milk, gives the details of two cases which indicate very clearly that the excessive use of wine by the nurse is dangerous for the child at her breast.

In the first case, observed by Charpentier, a three-weeks-old infant, whose nurse seemed to be in excellent condition, after thriving up to a certain date, began to be agitated and enervated upon each occasion when it was put to the breast; it ceased to sleep, had a reddened aspect and lost that contented expression of the child which is properly fed. And yet the nurse had an abundance of milk, very rich in globules. After several days, the child being then five weeks old, it exhibited a copious rash upon the face, the neck and portions of the trunk; the agitation which followed each application to the breast persisted and a real convulsive attack followed, which could not be explained by any of the causes usually operative in such cases. After an exceedingly careful investigation, it was finally discovered that the nurse, finding the large and vigorous infant capable of exhausting her supply of milk, which had already been furnished for nine months, had, in order to increase the quantity, drunk four bottles of wine daily. This amount she had tolerated so well, as not to excite the suspicions of her mistress. Charpentier concluding, hence, that the child was suffering from alcoholic intoxication, had the nurse placed under surveillance and limited daily to a half-bottle of wine, a bottle of beer and a litre or two of barley-water, with a nutritious diet. In a few days, the infant completely recovered its health, the agitation and convulsions ceased and the rash disappeared.

Verney cites a similar case where convulsions of the infant resulted from the alcoholic habits of the nurse, who drank from six to eight glasses of wine daily, and one during the night. The convulsions resisted bromide of potassium, musk, baths, calomel and belladonna, and yielded only when the nurse was restricted in the quantity of wine imbibed.

Excessive Cauterization of the Cervix Uteri.—J. Wallace. (*British Medical Journal*, 1877, Oct. 6.)—In our days the application of caustics to "an ulcer of the womb" has become such a routine practice that the following observations of Dr. W. may well merit our attention: "What are the immediate pathological results of weekly or bi-weekly applications of the nitrate of silver, which is the agent in most favor with practitioners, to the os tincæ? Inflammation and ulceration, lit up again and again by each application, followed in some instances by atrophy, and in others by hypertrophy of the cervix, with hard, painful cicatrices, and more or less contraction of the tissues and closure of the os, or even complete obliteration; endocervicitis and endometritis, the mucous lining of the cervical canal being more or less destroyed, and an ulcerated irregular surface remaining instead. Secondary functional derangements of the uterus follow, manifested first by dysmenorrhœa of a metrorrhagic character; and as the obstruction increases, regurgitation of the menstrual fluid takes place through the fallopian tubes, setting up ovarian, perimetritic, and general pelvic inflammations, with all their subsequent miseries.

The portio vaginalis uteri in some cases is so destroyed by caustics, that a hard, depressed cicatrix alone remains of it, and this is generally painful to the touch; the os is small, but not sufficiently so to account for the severe dysmenorrhœa. In such cases, the mucous membrane of the cervical canal is disorganized, and covered with uneven granulations, which bleed on the most gentle touch of the sound, and are so actually sensitive, that I have frequently found it necessary to give chloroform before it was possible to examine the condition of the uterus. In a case under my care lately, the canal was tortuous from these excessive granulations; yet, on passing the speculum, the mucous membrane of the cervix seemed free from all inflammatory irritation, and it was only by a minute examination of the canal of the cervix that it was seen to be denuded of healthy membrane, while a careful digital examination revealed to the touch irregularities marking the different cicatrices. Sexual intercourse could not be tolerated; uterine pain was ever present; and although she had been cauterized externally and internally by various practitioners during a period of several years, no relief was obtained. The cicatrices were clearly seen with the aid of a duck-bill speculum. This uterus has never been impregnated, yet it measured fully two and a half inches, although its vaginal portion had been destroyed to more than half its extent. Relatively, it was enlarged from menstrual obstruction. Free division of the cervix cured this case.

Painful cicatrices, atresia uteri, and loss of sexual desire are mentioned as the chief evil consequences of excessive cauterization. In conclusion, W. says: "Repeated cauterisation is not necessary to produce healing, for the severe ulceration produced by potassa fusa on the lips of the uterus will heal in from four to six weeks without any subsequent cauterization whatever. It is only necessary to direct the patient to use the vaginal douche daily, either warm water, carbolized water, sulpho-carbolate of zinc, or whatever astringent in solution that may be deemed suitable. The occasional passage of the sound during the healing process will obviate any tendency to atresia."

The treatment may be summarized thus: 1. "For painful cicatrices, divide the cervix freely; if that fail, remove them by excision. 2. For recent atresia, use rapid dilatation; for atresia of old standing, free division with the scissors and uterotome; simple medicated dressing afterwards, if there be endometritis. 3. The sound should be passed a week before each menstrual period for three or four months, to prevent reclosure."

THE ABSORPTION OF CERTAIN MEDICAMENTS BY THE PLACENTA AND THEIR ELIMINATION BY THE URINE OF NEW-BORN CHILDREN.—Porack. (*Journal de Thérapeut.*, Sept. 25, 1877.) The substance first employed by the author was the iodide of potassium, because of the readiness with which it may be recognized in the urine. He administered the potassic salt a few moments or hours before delivery, and examined several times consecutively the urine of the child in order to establish its elimination.

The following conclusions were obtained from his first experiments:

1. In doses of twenty-five centigrammes, the iodide of potassium constantly passes into the urine of the new-born child, more promptly when the dosage is increased.

2. More than half an hour is requisite for the drug to traverse the placenta; after an interval of forty minutes, the characteristic reaction can always be detected in the urine of the new-born child.

3. When the urine is collected immediately after birth, and the reaction is compared with that obtained in urine collected some hours after, the first reaction is always the more feeble. This reaction is never obtained in the urine of the still-born child. The eliminating activity is therefore developed only after birth. Before delivery the placenta is an organ of absorption and elimination for medicaments temporarily penetrating the fœtal organism.

4. When twenty-five centigrammes of the iodide of potassium are given to a woman in labor, the quantity absorbed by the placenta is small, and its elimination by the urine of the new-born child rapid.

5. When fifty centigrammes (and particularly one gramme) are administered to a woman, elimination by the kidneys of the child is always much more retarded than by those of the mother. While it is generally true that the total quantity of the iodide is eliminated by the mother in thirty-six hours, it is not rare to find the elimi ation by the child incomplete by the fourth day. In two cases it was not completed by the fifth and sixth days.

The importance of the slow elimination of the medicament by the child after treatment of the mother in labor, or of the nurse, lies in the fact that, if the elimination by the former is less rapid, the accumulation is greater, and thus the child more speedily presents the phenomena of intoxication.

Relations of Pregnancy to Surgical Affections (*Centralbl. f. Chirurg.*, 1877, No. 26; from *Bull. de la Soc. de Chirurgie*).—Verneuil, after alluding to the vast contributions made to surgical science within the last few years, remarks upon the necessity of having certain doubtful points cleared up, among others that concerning the reciprocal influence of pregnancy and surgical affections. To the solution of the question Verneuil contributes full notes of nine observations, which cannot be quoted for want of space. The conclusions which he draws are formulated as follows: Pregnancy and surgical affections (traumatic or of other kinds) may occur at the same time without in any way influencing each other. On the other hand, surgical affections may give rise to death of the fœtus, or miscarriage. Pregnancy, moreover, although in itself pursuing a normal course, may give rise to surgical affections (as for instance the occurrence of phlegmonous dacrocystitis), modify the course of others unfavorably, and give a peculiar unhealthy look to wounds of all kinds. In addition, concludes Verneuil, the unfavorable course of local affections during pregnancy may react upon the latter in the same manner. This unfavorable condition of local affections is so apt to change for the better after delivery, that Verneuil thinks the question may sometimes arise whether the production of premature labor might not be desirable.

Etiology of Fibroid Tumor of the Uterus.—F. Englemann, (*Centralbl. f. Chirurgie*, No. 27, 1877 from *Zeitschr. f. Geburtsh. und Gynäkologie*, Bd. i. Hft. i. p. 130) notes three hundred and sixty-two cases of uterine fibroid, of which sixty-five have come under his own notice, and the remainder under that of his father, and compares them with the observations of other writers. Married women, according to E.'s statistics, are more frequently the subjects of this affection than the unmarried, 5.1 to 1. (According to Winckel the proportion is 9 to 7.3.) Both observers find the affection most frequent between the ages of forty and fifty. In nearly one-half the cases, the earliest symptoms appeared between the thirtieth and fortieth years. Among the married women 26 per cent. were sterile. Among the married women who had borne one or more times before the appearance of the symptoms, eleven never conceived afterwards, seven underwent one or more abortions, and twelve bore living children.

By taking advantage of especial opportunities, E. found among his sixty-five cases, in thirty-two no positive data; in the remaining thirty-three, he thought himself justified in twenty-six cases in attaching importance to the assertions of the patients relative to the origin of the disease. Together with abortion, inflammation of the abdominal region (six), and difficult labor (three), typhus, strain, fall, catching cold, dancing, climbing, agitation of the mind, and "diseases" were believed to be causes of the fibroid development. E. found the baths of Kreutznach effectual in removing the growth in a number of instances.

